# The Effect of VPA on Increasing Radiosensitivity in Osteosarcoma Cells and Primary-Culture Cells from Chemical Carcinogen-Induced Breast Cancer in Rats

**DOI:** 10.3390/ijms18051027

**Published:** 2017-05-10

**Authors:** Guochao Liu, Hui Wang, Fengmei Zhang, Youjia Tian, Zhujun Tian, Zuchao Cai, David Lim, Zhihui Feng

**Affiliations:** 1 Department of Occupational Health and Occupational Medicine, School of Public Health, Shandong University, Jinan 250012, China; 201514107@mail.sdu.edu (G.L.); sduwanghui03@163.com (H.W.); fengmeizhang2003@sdu.edu.cn (F.Z.); 201514110@mail.sdu.edu (Y.T.); tianzhujun_mercy@163.com (Z.T.); 201614266@mail.sdu.edu (Z.C.); 2 Flinders Rural Health South Australia, Victor Harbor, SA 5211, Australia; david.lim@flinders.edu.au or c113.lim@qut.edu.au

**Keywords:** VPA, DNA double-strand breaks, radiosensitivity, DNA repair, U2OS, chemical carcinogen (DMBA)-induced tumor

## Abstract

This study explored whether valproic acid (VPA, a histone deacetylase inhibitor) could radiosensitize osteosarcoma and primary-culture tumor cells, and determined the mechanism of VPA-induced radiosensitization. The working system included osteosarcoma cells (U2OS) and primary-culture cells from chemical carcinogen (DMBA)-induced breast cancer in rats; and clonogenic survival, immunofluorescence, fluorescent in situ hybridization (FISH) for chromosome aberrations, and comet assays were used in this study. It was found that VPA at the safe or critical safe concentration of 0.5 or 1.0 mM VPA could result in the accumulation of more ionizing radiation (IR)-induced DNA double strand breaks, and increase the cell radiosensitivity. VPA-induced radiosensitivity was associated with the inhibition of DNA repair activity in the working systems. In addition, the chromosome aberrations including chromosome breaks, chromatid breaks, and radial structures significantly increased after the combination treatment of VPA and IR. Importantly, the results obtained by primary-culture cells from the tissue of chemical carcinogen-induced breast cancer in rats further confirmed our findings. The data in this study demonstrated that VPA at a safe dose was a radiosensitizer for osteosarcoma and primary-culture tumor cells through suppressing DNA-double strand breaks repair function.

## 1. Introduction

Among other methods, chemotherapy and radiotherapy are generally prescribed for the treatment of cancers, and such DNA-damaging cytotoxic therapies remain the main treatment of cancers such as osteosarcoma and breast cancer. However, with time, tumor cells develop mechanisms of resistance to such treatments. Recently, considerable attention has been on researching effective strategies to understand and develop means of decreasing tumor cellular sensitization and resistance to DNA-damaging agents. Histone deacetylase (HDAC) was identified as a promising therapeutic target for cancer treatment as it plays a central role in chromosome structural remodeling and gene-transcriptional regulation, with altered expression and mutation of HDAC linked to tumor development and occurrence [1]. Eighteen mammalian HDACs have been identified so far [2], and have been subdivided into four different classes based on their homology with yeast HDACs. HDAC inhibitors (HDACi), such as the anticonvulsant drug valproic acid (VPA), have been identified as neoadjuvant to chemotherapy and radiotherapy [3]. The VPA-induced sensitization of tumor cells has been attributed to its effect on HDAC-dependent transcriptional repression and hyperacetylation of histones, which resulted in the differentiation of tumor cells and increased both apoptotic and non-apoptotic cell death [4,5]. Previous studies have demonstrated that VPA downregulated key proteins such as BRAC1, RAD51, Ku70, Ku80, and prolonged radiation-induced repair protein foci such as γH2AX and 53BP1 in tumor cells [1,6,7,8,9]. This is important as DNA-damaging cytotoxic therapies are intended to induce DNA-double strand breaks (DSBs). We have previously demonstrated that VPA increased the radiosensitivity of breast cancer cells through the disruption of both BRAC1-Rad51-mediated homologous recombination and Ku80-mediated non-homologous end-jointing [7]. However, some results indicated that radiotherapy was largely ineffective in osteosarcoma [10,11], so it would be very interesting to investigate whether VPA could enhance the radiosensitivity of osteosarcoma cells, which may be helpful for osteosarcoma treatment in medical clinics.

The safe blood concentration of VPA for the treatment of epilepsy in clinic is 50–100 μg/mL, which is equal to 0.3–0.8 mM. Based on this information, our study selected 0.5 mM and 1.0 mM as a safe dose and a critical safe dose, respectively, for the treatment of epilepsy in clinic to explore the effect of VPA on radiosensitivity and its mechanism in osteosarcoma cells (U2OS cell line) and primary-culture cells from the tissue of chemical carcinogen (DMBA)-induced breast cancer in rats. Our results clearly suggest that a safe dose of VPA could induce more DSBs in both working systems in response to DNA damage induced by IR, and increase radiosensitivity and genetic instability in the cells by disrupting DNA repair function.

## 2. Results

### 2.1. Effects of VPA on DNA-Double Strand Breaks (DSB) in Osteosarcoma Cells

**Figure 1:**
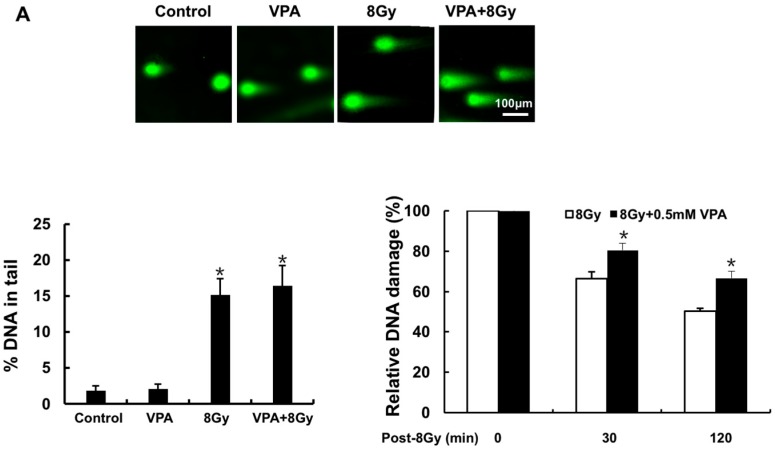

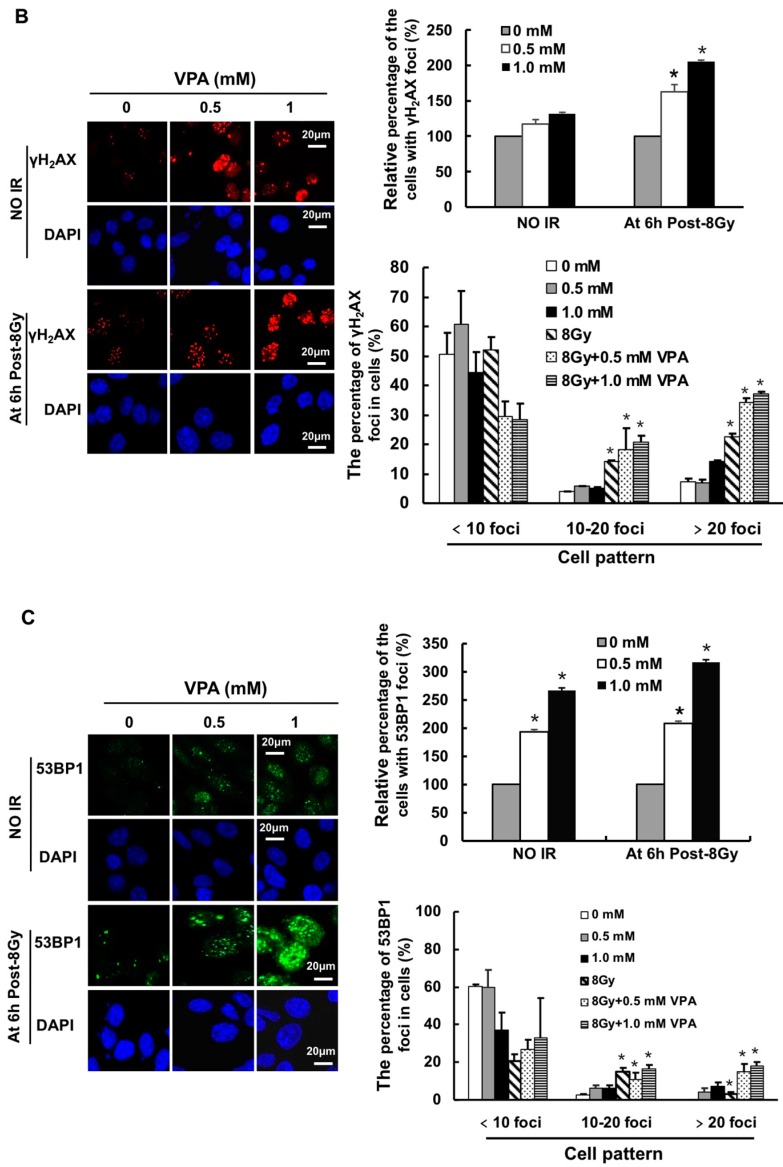
VPA can cause the accumulation of more IR-induced DNA DSBs in U2OS cells. (**A**) 0.5 mM VPA-treated and untreated cells before and after 8 Gy treatment are presented in the images from comet assay (**upper**), and the olive moment was further analyzed (**lower left**); after correcting the data, the relative olive moment at 0, 30, and 120 min post-IR was exhibited in the cells (**lower right**); (**B**) The images represent the γH2AX foci formation in the cells treated with 0.5 or 1.0 mM VPA before and after IR (8 Gy) treatment (**left**), and the percentage of cells with γH2AX foci formation in each group was calculated (**right upper panel**, the cell with >10 foci was called positive and counted), also the percentage of cells with different patterns divided by the number of foci per nucleus in each group estimated (**right lower panel**); (**C**) The images represent 53BP1 foci formation in the cells treated with 0.5 or 1.0 mM VPA before and after IR (8 Gy) treatment (**left**), and the percentage of cells with 53BP1 foci formation in each group was calculated (**right upper panel**, the cell with >10 foci was called positive and counted), the percentage of cells with different patterns divided by the number of 53BP1 foci per nucleus in each group estimated (**right lower panel**). 4′,6-diamidino-2-phenylindole (DAPI) was used for nuclear staining. 2.5 × 10^4^ U2OS cells were seeded on the chamber in immunofluorescence assays. Each data point in the graphs was from three independent experiments (mean ± SD). *p*-Values were calculated by Student’s *t*-test (* *p* < 0.05).

To quantify the effects of VPA on the DSB using neutral comet assay, a U2OS cell line was pretreated with 0.5 mM VPA and subjected to 4 Gy ionizing radiotherapy (IR). With and without IR, there was no statistically significant difference between the DNA-tail of VPA versus untreated-control; however, visually it does appear that cell-lines pretreated with VPA exhibited a longer DNA tail (Figure 1A upper). VPA + IR had statistically more relative DSB compared to post-IR at both 30- and 120-min (Figure 1A lower right, *p* < 0.05). The findings inferred that VPA caused the accumulation of more IR-induced DSB in osteosarcoma cells, and a slower recovery of DSB in a time-dependent manner.

To triangulate the above-mentioned findings, DSB-induced histone H2AX phosphorylation on serine 139 (γH2AX) formation [12,13,14], and p53 binding protein 1 (53BP1)—the markers for DSB [15,16,17,18]—were analyzed. The U2O2 cell line was pretreated with 0.5 mM and 1 mM VPA for 24 h before being subjected to 8 Gy radiation. At 6 h post-IR, the immunofluorescence staining showed that safe doses of VPA at 0.5 and 1.0 mM induced an increased γH2AX foci formation when compared to the control group (Figure 1B). The foci’s size and density in the VPA-pretreated group appeared larger and brighter than the IR-alone group (Figure 1B left). The relative percentage of cells with γH2AX foci increased by 1.6 and 2.1-fold, respectively (Figure 1B right upper, *p* < 0.05). We further analyzed the above data in another way by categorizing the cells containing γH2AX foci into three patterns according to the number of foci in each cell: <10, 10–20, and >20 (Figure 1B right lower). There was a positive association between the number of γH2AX foci and VPA-treatment (*p* < 0.05). Likewise, the relative ratio of U2O2 cells with 53BP1 foci pretreated with 0.5 mM and 1.0 mM VPA increased by 2.1 and 3.2-fold, respectively (Figure 1C left and right upper, *p* < 0.05). Similarly, we observed appositive association between number of 53BP1 foci and VPA-treatment (Figure 1C right lower, *p* < 0.05).

**Figure 2:**
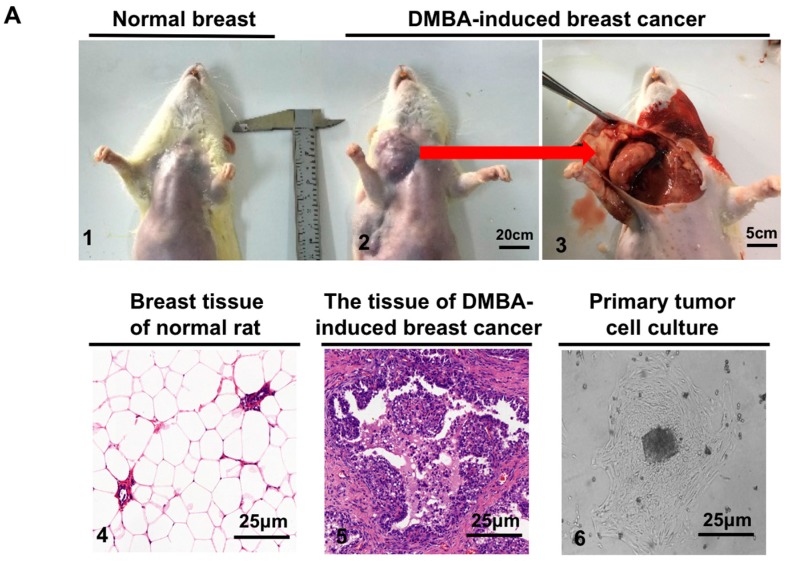

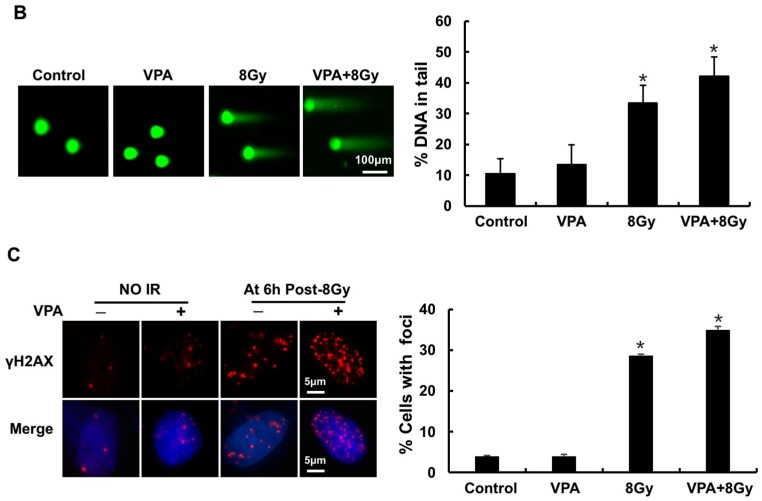
The effect of the combination of VPA and IR on primary-culture cells of breast cancer tissue in rats. (**A**) Normal breast (**1**), and DMBA-induced breast cancer (**2** and **3**) of rats under gross observation; HE staining for the morphology of normal tissue (**4**) and DMBA-induced breast cancer (**5**); the primary cell culture of breast cancer tissue (**6**); (**B**) The untreated and 0.5 mM VPA-treated cells are presented in the images from comet assay before and after 8 Gy treatment (**left**), and the olive moment was further analyzed (**right**); (**C**) The images represent the γH2AX foci formation in the cells treated with 0.5 mM VPA at 6 h post-IR, “+” and “−” indicated whether VPA was added in the groups (**left**); the percentage of cells with γH2AX foci formation in each group was calculated (**right**, the cell with >10 foci were called positive and counted). DAPI was used for nuclear staining. Each data point in the graphs was from three independent experiments (mean ± SD). *p*-Values were calculated by Student’s *t*-test (* *p* < 0.05).

To confirm the radiosensitization effect of VPA on the tumor cells, a model of chemical carcinogen (DMBA)-induced breast cancer in SD rats was established to obtain primary-culture tumor cells. Rats at 50 days old were gavaged DMBA to induce tumor formation around the rats’ nipples, which was detached from skin around 90 days after DMBA administration (Figure 2(A1–A3)). The morphological structure of the tissue was observed by hematoxylin-eosin (HE) staining. Figure 2(A4) shows that the structure of breast tissue in normal rats in contrast with a large number of hyperplasia cells in the DMBA-induced breast cancer tissue (Figure 2(A5)), indicating that breast cancer in rats was successfully induced by this chemical carcinogen. Primary-culture tumor cells were then obtained from this breast cancer tissue (Figure 2(A6)). Two methods of neutral comet assay and γH2AX foci were used to test the radiosensitivity effect of VPA on the cells. At 0 min post-IR, the combination of 0.5 mM VPA and 8 Gy significantly increased the olive moments in the cells when compared with IR alone (Figure 2B, *p* < 0.05), suggesting that VPA could induce more IR-caused DSBs. Additionally, similar results were found via the γH2AX foci formation assay. For the combined treatment group, at 6 h post-IR treatment, the percentage of primary-culture tumor cells containing γH2AX foci was obviously higher than that of the IR alone group (Figure 2C, *p* < 0.05), confirming that VPA can lead to more DSBs damage in response to IR treatment. The above-mentioned results clearly revealed that VPA was a radiosensitizer not only for the tumor cell line, but also for the primary-culture tumor cells.

### 2.2. Effects of VPA on Radiosensitivity of Tumor Cells

**Figure 3:**
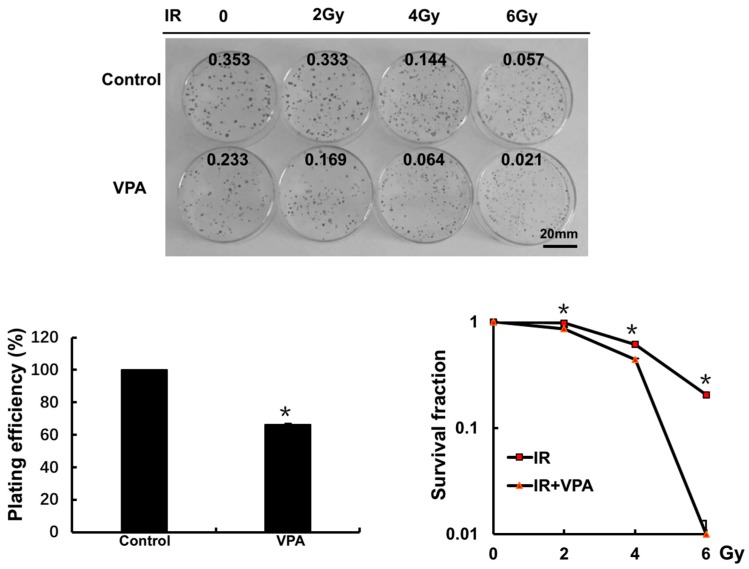
VPA can increase the radiosensitivity in U2OS cells. Plating Efficiency (PE) was presented in untreated and VPA-treated cells (**lower left**) A clonogenic survival assay was used to detect survival in the cells treated with different doses of IR (0, 2, 4, or 6 Gy) and the combination of 0.5 mM VPA with different doses of IR (**lower right**). Each data point in the graphs was from three independent experiments (mean ± SD). *p*-Values were calculated by Student’s *t*-test (* *p* < 0.05).

To understand whether the above-observed VPA effects on DSB may be associated with cellular radiosensitivity, a clonogenic survival assay was employed. The U2O2 cell line was pretreated with 0.5 mM VPA and then exposed to 0, 2, 4, and 6 Gy radiation, respectively. The cells were cultured for a further 14 days, and the clonogenic colonies were stained and counted (Figure 3 upper). The VPA-treated group showed a decreased survival fraction when compared to the control group (Figure 3 lower left, *p* < 0.05). After the survival fraction of IR and the combination of VPA with IR was corrected by the corresponding control group, the data showed that there was a significant decrease in all combinations of VPA and IR groups when compared with relative IR groups (Figure 3, bottom right, *p* < 0.05). Additionally, the size of the colonies in all groups of the combination of VPA and IR were smaller in appearance than those of IR alone (Figure 3, top). The findings suggest that VPA increased the radio-sensitivity of tumor cells and suppressed tumor cell growth in response to DNA damage.

### 2.3. VPA at Safe Dose Can Arise the Dysfunction of DNA Repair

**Figure 4:**
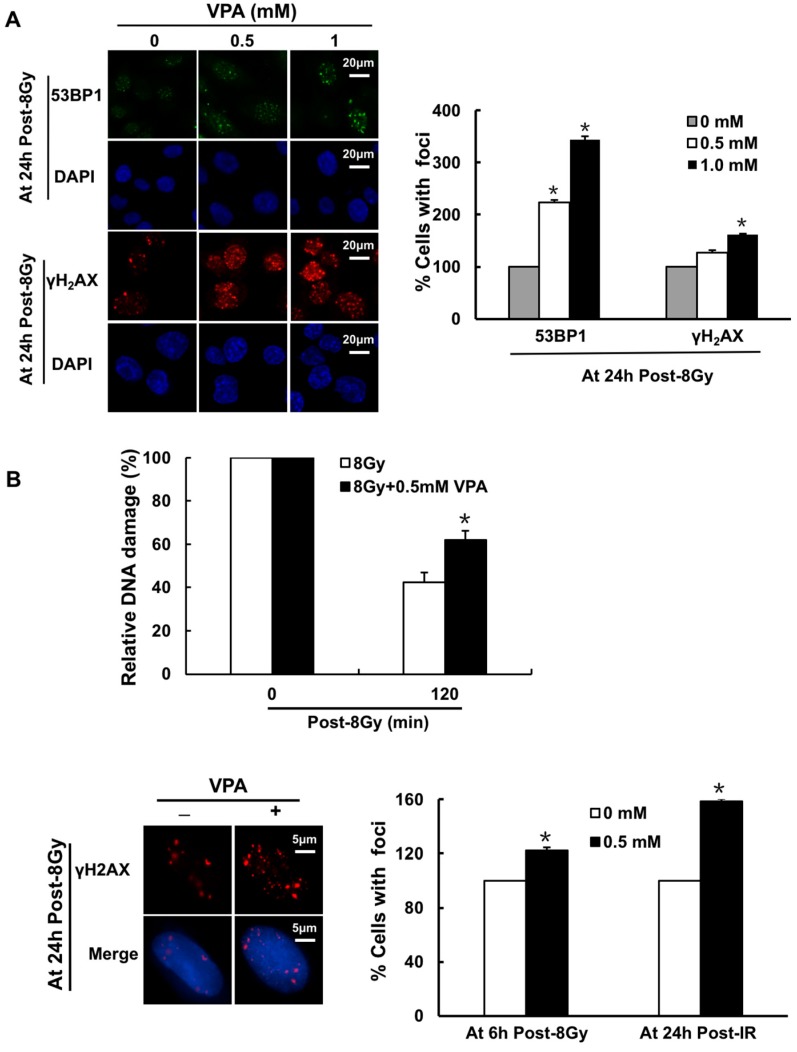

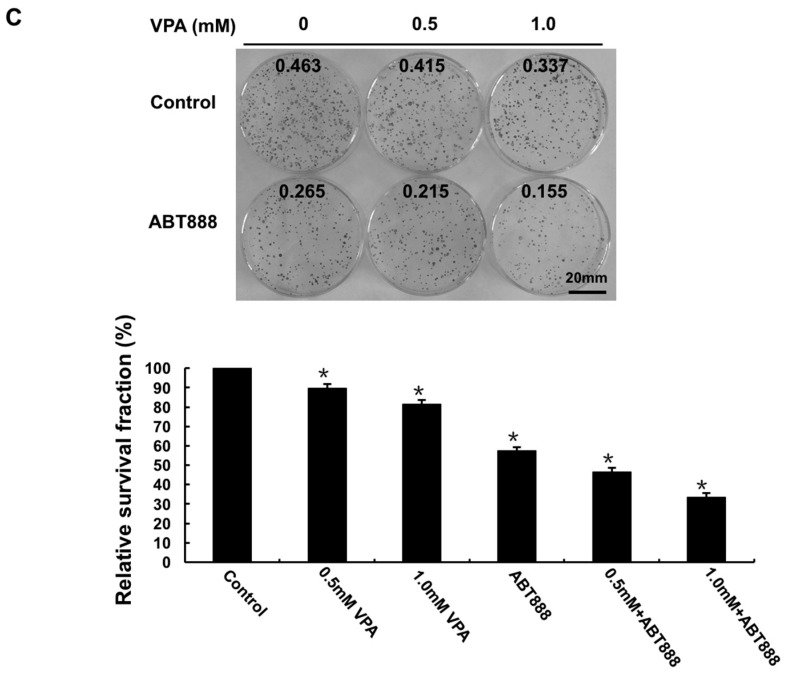
VPA at a safe dose can lead to the dysfunction of DNA repair function. (**A**) The images represent γH2AX and 53BP1 foci formation in U2OS cells treated with 0.5 or 1.0 mM VPA at 24 h post-IR treatment (**left panel**), and the percentage of cells with γH2AX foci or 53BP1 foci formation in each group was calculated (**right panel**, the cell with >10 foci was called positive and counted). DAPI was used for nuclear staining; (**B**) The relative DNA damage in primary-culture tumor cells at 120 min post-IR was analyzed by comet assay (**upper**), and γH2AX foci formation in primary-culture tumor cells at 24 h post-IR was presented，“+” and “−“ indicated whether VPA was added in the groups (**lower left**) and calculated (**lower right**); (**C**) The clonogenic survival assay was used to detect survival in the U2OS cells treated with the combination of 0.5 or 1.0 mM VPA with 10 μM ABT888 (PARPi). Each data point in the graphs was from three independent experiments (mean ± SD). *p*-Values were calculated by Student’s *t*-test (* *p* < 0.05).

There is a biphasic DNA repair mechanism post-IR: the early (0–6 h post-IR), and late (6–24 h post-IR) phases. Congruent with previous study [7], immunofluorescence staining of γH2AX and 53BP1 foci was utilized to study the mechanisms of VPA-induced radio-sensitivity in U2O2 cells 24 h post-6 Gy IR treatment. There was a statistically significant increase in the percentage of cells with γH2AX (0.5 mM: 1.27-fold, *p* < 0.05; 1 mM: 1.62-fold, *p* < 0.05) and 53BP1 (0.5 mM: 2.24-fold, *p* < 0.05; 1 mM: 3.43-fold, *p* < 0.05) foci with VPA-treatment (Figure 4A). These findings indicated that VPA may impact the repair ability of DNA in the late phase.

Next, primary-culture tumor cells from breast cancer tissue were used to test DNA repair activity. After the data was corrected with the corresponding control group at 120 min post-IR, the olive moments in the cells treated with a combination of VPA and 8 Gy were significantly higher when compared with IR alone (Figure 4B upper, *p* < 0.05). Similar results were also found via a γH2AX foci formation assay, at both 6 h and 24 h post-IR treatment, the percentage of primary-culture tumor cells containing γ-H2AX foci in the combined treatment group was obviously higher than that of the IR alone group (Figure 4B lower, *p* < 0.05). Thus, the results suggested that DNA repair activity was suppressed by VPA as a late response to IR treatment.

Synthetic lethality (SL) was first defined as a genetic combination of mutations in two or more genes that leads to cell death, whereas a mutation in any one of the genes does not [19,20]. The theory of SL in the DNA damage repair field has recently grown in popularity with the finding that poly (adenosine diphosphate (ADP)-ribose) polymerase inhibitors (PARPi) are specifically toxic to BRCA1 or BRCA2-associated homologues recombination (HR)-defective cells [21,22]. We speculated that the combination of VPA with PARPi would cause cell death if the VPA could inhibit HR function; thus, a clonogenic survival assay was used to study the effect of VPA and a typical poly ADP-ribose polymerase inhibitor, ABT888, on cell survival. Figure 4C demonstrates that 10 μM ABT888 alone, VPA alone, and VPA + ABT888 significantly reduced the relative survival fraction. The combination of 1 mM VPA and ABT888 had the lowest relative survival fraction (33.51%). The results indicated that the actions of VPA on suppressing tumor cell growth may be through its effect on DNA repair functions.

### 2.4. Effects of VPA on Chromosome Aberrations

**Figure 5:**
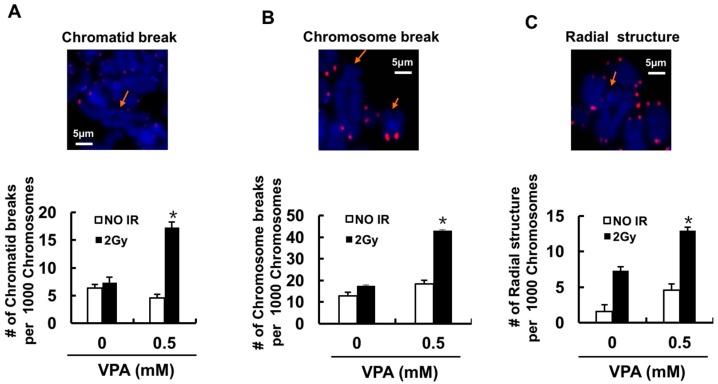
The effect of VPA on chromosome aberrations in U2OS cells. The untreated or VPA-treated cells were irradiated by 2 Gy, and the frequencies of IR-induced chromosome aberrations were analyzed. Fluorescence in situ hybridization using a telomeric probe is indicated in pink, and chromosomes were stained with DAPI in blue. Fifty metaphases for each sample were scored. Chromatid break (**A**), chromosome break (**B**), or radial structure (**C**) were presented and pointed by the arrow. “#”in each graph was indicated the number of the break. Each data point in the graphs was from three independent experiments (mean ± SD). *p*-Values were calculated by Student’s *t*-test (* *p* < 0.05).

To test the effects of VPA on genomic stability, Q-FISH was utilized for the analysis of chromosome aberrations. Figure 5 showed no statistical difference in the number of chromatid and chromosome breaks between control and IR-treatment groups, whilst IR increased the number of radical structure from 1.59 per 1000 chromosomes to 7.34 (*p* < 0.05). The pre-treatment with 0.5 mM VPA significantly increased the number of chromatid breaks (4.57 per 1000 chromosomes to 17.24, *p* < 0.01), chromosome breaks (18.27 per 1000 chromosomes to 43.10, *p* < 0.01), and radical structure (4.57 per 1000 chromosomes to 12.93, *p* < 0.01). The findings demonstrated that VPA could lead to genomic instability through its effects on chromosome aberrations in response to IR.

## 3. Discussion

It has been increasingly proposed that the effect of HDAC inhibitors in the radiosensitization of tumor cells occurred via their effects on the DNA repair pathway [7,23]. Our previous results demonstrated that safe doses of VPA can radiosensitize the breast cancer cells by affecting both DNA DSB repair pathways, as well as decrease the frequency of homologous and non-homologous end joining [7,24,25]. In this study, we investigated whether safe concentrations of VPA could induce more IR-induced DSBs and inhibit cell survival in vivo using osteosarcoma cells and chemical-induced breast cancer cells. The use of primary tissue culture was important as this model mimicked the development of human primary tumors in situ. Our findings demonstrated that VPA did induce the radiosensitization of tumor cells and the effects of this HDAC inhibitor operates through suppressed DNA repair and associated genomic instability. Together with previous reports of VPA augmented radiation-induced apoptosis through targeted activity on BRCA1, Rad51 and Ku80 proteins [7,26,27], this study advanced the proposal for the use of VPA as a neoadjuvant to radiotherapy for cancer treatment.

As DNA repair functions, such as HR and Non-Homologous End Joining (NHEJ), are an important mechanism for HDACi-radiosensitization in tumor cells, some results indicated that the effect of HDACi on them was inconsistent, which may be relative to the HDACi used as in the study as our data demonstrated that VPA could decrease the frequency of HR and NHEJ in breast cancer cells [7,24]. Other reports also found that both NHEJ and HR rates decreased in the presence of butyrate, it was estimated that NHEJ decreased by 40% and HR decreased by 60% [28]. However, it was observed that 5 mM or 10 mM VPA could enhance HR after treatment for 24 h in Chinese hamster ovary (CHO) 3–6 cells [25], and Suberoylanilide hydroxamic acid (SAHA) and Trichostatin A (TSA) could only increase NHEJ activity but did not change the HR frequency in HeLa cells [7,28]. HDACi can affect several key proteins in DSBs repair such as p53, BRCA1, RAD51, and Ku80. Our previous report pointed out that VPA could disrupt HR and NHEJ through targeting the activity of BRCA1, Rad51, and Ku80 [7], may enhance radiation-induced apoptosis and serve as a radiosensitizer in a p53-dependent manner in colorectal cancer cells [26], and downregulate both protein expression and foci accumulation of BRCA1 and RAD51 in LNCaP and DU-145 cells [27,29]. Vorinostat and TSA could also attenuate upregulation of Ku80 and DNA-PKcs in prostate and colon cancer cells [29]. SAHA attenuated radiation-induced Rad51 and Ku80 protein expression in two sarcoma cell lines (KHOS-24OS, SAOS2) [30]. 

In this study, we also used primary-culture cells of chemical-induced breast cancer model to detect VPA-induced radiosensitivity. This model successfully mimicked the development of human primary tumor by a chemical carcinogen, DMBA. The results indicated that VPA can radiosensitize tumor cells through inhibiting DNA repair function, which provides strong evidence in support of the effects of VPA on radiosensitivity and exhibited a worthy implication for the study of its clinical trial and preclinical study. However, we still need to further explore how VPA influences tumor radiosensitivity in vivo in this primary tumor model.

Therefore, sensitization of tumor cells via inhibition of the DNA damage repair response may contribute a broader and more meaningful strategy to improve radio-therapy efficacy for tumor patients.

## 4. Materials and Methods

### 4.1. Materials

VPA was purchased from Sigma (St. Louis, MO, USA). The concentration of 0.5 mM and 1.0 mM were chosen as a safe and critical dose, respectively, as informed by previous work [7].

### 4.2. Cell Line

The U2OS osteosarcoma cell line was obtained from Maria Jesin’s Lab in Developmental Biology Program, Memorial Sloan-Kettering Cancer Centre, New York, NY, USA. The U2OS cell line was cultured in DMEM medium (Gibco, Carlsbad, CA, USA) with 10% fetal bovine serum (Gibco, Carlsbad, CA, USA), 100 μg/mL streptomycin and 100 units/mL penicillin (Sigma). The cell line was grown at 37 °C with a humidified environment of 5% carbon dioxide. The cell line was treated with VPA and 10 μM ABT888 (poly ADP-ribose polymerase inhibitor, Active Biochemicals, Hong Kong, China) for in vitro clonogenic assay.

### 4.3. Tissue Culture and Animal Husbandry

Female Sprague-Dawley (SD) rats were purchased from Pengyue Laboratory Animal Co. Ltd. Jinan, China. The studies of animal tissue were performed in accordance with the requirements of the Shandong University Human and Animal Ethics Research Committee (The project identification code is 81472800, the date of approval was 3 March 2014 issued by the ethics committee review board of prevention medicine in Shandong University of China). All rats were housed in a specific-pathogen-free environment, at a temperature of 23 ± 1 °C. The lights were at a daily rhythm of 12 h and the SD rats were fed fresh food and water ad libitum throughout the experiment. The care of the animals was in accordance with the relevant Chinese laws and guidelines used for experimentation and scientific purposes. Breast tumors were induced in 50 day old female SD rats (weighted 150 ± 15 g; *n* = x) by a single administration of 20 mg/mL DMBA (7,12-dimethylbenzanthracene, Sigma, St. Louis, MO, USA) dissolved in sesame oil by oral gavage. The rats were palpated twice weekly for tumors. The rats where tumor burden was approximately 10% of total body weight were killed on day 90. All other rats were euthanized 20 weeks after the administration of DMBA.

Rats were injected intraperitoneal with 1 mL chloral hydrate (Sigma, St. Louis, MO, USA) for anesthesia, then sodium sulfide (Sigma, St. Louis, MO, USA) was used for unhairing. Breast tumor induced by DMBA in rats was sterilely isolated and mechanically dissociated into approximately 2 mm^3^ of tissue was utilized. The tumor specimens were put onto P60 dishes and incubated with 20% fetal bovine serum and cultured at 37 °C with a humidified environment of 5% carbon dioxide for primary cell culture. Around 10 days, the cells grew from tissue and the cells were used for relative study.

The morphological structure of the tissue was observed by HE staining. Figure 2(A4) showed that the structure of breast tissue in normal rats had a few duct and acinus; In contrast, a large number of hyperplasia cells were found in the breast cancer tissue and the cell arrangement in the tumor tissue was also part of the disorder (Figure 2(A5)), indicating that the breast cancer in these rats was successfully induced by this chemical carcinogen. The primary culture tumor cells were obtained from the breast cancer tissue (Figure 2(A6)).

### 4.4. Clonogenic Survival Assay

The clonogenic survival assay was described in our previous publications [7,31]. In brief, the U2OS cells and primary culture cells from breast cancer tissue in rats were treated with 2, 4, or 6 Gy of IR using a Siemens Stabilipan 2 X-ray generator (Qilu Hospital, Jinan, China) operating at 250 kVp 12 mA at a dose rate of 2.08 Gy/min. For the combination group, the cells were pretreated with 0.5 mM for 24 h, then further irradiated with different doses. The number of cell colonies (≥50 cells per clone) was counted and cell survival was presented by the survival fraction (SF): SF = (the number of clones/seeded cells)/plating efficiency (PE).

For clonogenic survival assay in the cells treated with both VPA and ABT888, the cells were pretreated with 0.5 or 1.0 mM VPA for 24 h, and then 10 μM ABT888 was added for a further 24 h incubation. The SF in each group was also analyzed.

### 4.5. Quantitative Fluorescence In Situ Hybridization (Q-FISH) for Chromosomal Aberration Analysis

As described in Reference [31,32,33], the 2 Gy treated cells with the pretreatment by VPA for 24 h and culture were incubated for 20 h before 0.05 g/mL colcemid (Gibco, Carlsbad, CA, USA) was added for a further 4 h incubation to obtain metaphase cells.

### 4.6. Comet Assay for DNA DSBs

The neutral comet assay was performed using the Trevigen Comet Assay kit and was described in our recent publication [7]. Simply speaking, the comet tail in VPA-treated, or untreated cells at 0, 60, and 120 min post-8 Gy were analyzed. For the comet assay used to examine comet tail in the cells at 0 min post-IR, VPA-treated or untreated cells were on ice during the whole irradiation process to allow the cell have minimum chance to repair damaged DNA. At this time point, whole DNA DSBs in the cells were presented. However, for the comet assay of cells at 60 and 120 min post-IR, VPA-treated or untreated cells did not require ice. Other steps of the comet assay were done in accordance with the standard procedures provided by the manufacturer (Trevigen Company, Gaithersburg, Montgomery County, MD, USA).

### 4.7. Immunofluorescence Assay of γH2AX and 53BP1

The cells were pretreated by VPA for 24 h and further irradiated. Then treated and untreated cells were rinsed with phosphate buffer saline (PBS) and fixed with paraformaldehyde. Cells were washed with PBST buffer (PBS + 0.2% Triton X-100), then blocked with 10% serum for 1 h and incubated with a primary antibody of γH2AX (Ser139, clone JBW301, Millipore, Darmstadt, Germany), or 53BP1 (NB100-304, NOVUS) overnight at 4 °C. The cells were further incubated with a secondary antibody of AlexaFluor 594-labeled goat anti-mouse I gG, or AlexaFluor 488-labeled chicken anti-rabbit (Thermo Fisher, Waltham, MA, USA) at a 1:300 dilution for 1 h in the dark after washing with PBST buffer, then stained with DAPI for nucleus [7,31].

### 4.8. Statistical Analysis

Results are expressed as means ± standard deviation for the groups. Data were analyzed by independent sample *t*-test. *p* < 0.05 indicated a statistically significant difference.

## References

[1] Groselj B., Sharma N.L., Hamdy F.C., Kerr M., Kiltie A.E. (2013). Histone deacetylase inhibitors as radiosensitisers: Effects on DNA damage signalling and repair. Br. J. Cancer.

[2] Mawatari T., Ninomiya I., Inokuchi M., Harada S., Hayashi H., Oyama K., Makino I., Nakagawara H., Miyashita T., Tajima H. (2015). Valproic acid inhibits proliferation of HER2-expressing breast cancer cells by inducing cell cycle arrest and apoptosis through Hsp70 acetylation. Int. J. Oncol..

[3] Elbadawi M.A.A., Awadalla M.K.A., Hamid M.M.A., Mohamed M.A., Awad T.A. (2015). Valproic Acid as a Potential Inhibitor of Plasmodium falciparum Histone Deacetylase 1 (PfHDAC1): An in Silico Approach. Int. J. Mol. Sci..

[4] Munster P., Marchion D., Bicaku E., Schmitt M., Lee J.H., DeConti R., Simon G., Fishman M., Minton S., Garrett C. (2007). Phase I trial of histone deacetylase inhibition by valproic acid followed by the topoisomerase II inhibitor epirubicin in advanced solid tumors: A clinical and translational study. J. Clin. Oncol..

[5] Marchion D.C., Bicaku E., Daud A.I., Sullivan D.M., Munster P.N. (2005). Valproic acid alters chromatin structure by regulation of chromatin modulation proteins. Cancer Res..

[6] Makita N., Ninomiya I., Tsukada T., Okamoto K., Harada S., Nakanuma S., Sakai S., Makino I., Kinoshita J., Hayashi H. (2015). Inhibitory effects of valproic acid in DNA double-strand break repair after irradiation in esophageal squamous carcinoma cells. Oncol. Rep..

[7] Luo Y., Wang H., Zhao X.P., Dong C., Zhang F.M., Guo G., Guo G.S., Wang X.W., Powell S.N., Feng Z.H. (2016). Valproic acid causes radiosensitivity of breast cancer cells via disrupting the DNA repair pathway. Toxicol. Res. UK.

[8] Van Oorschot B., Granata G., Di Franco S., ten Cate R., Rodermond H.M., Todaro M., Medema J.P., Franken N.A.P. (2016). Targeting DNA double strand break repair with hyperthermia and DNA-PKCS inhibition to enhance the effect of radiation treatment. Oncotarget.

[9] Chinnaiyan P., Cerna D., Burgan W.E., Beam K., Williams E.S., Camphausen K., Tofilon P.J. (2008). Postradiation sensitization of the histone deacetylase inhibitor valproic acid. Clin. Cancer Res..

[10] Mamo T., Mladek A.C., Shogren K.L., Gustafson C., Gupta S.K., Riester S.M., Maran A., Galindo M., van Wijnen A.J., Sarkaria J.N. (2017). Inhibiting DNA-PKCS radiosensitizes human osteosarcoma cells. Biochem. Biophys. Res. Commun..

[11] Zuch D., Giang A.H., Shapovalov Y., Schwarz E., Rosier R., O’Keefe R., Eliseev R.A. (2012). Targeting radioresistant osteosarcoma cells with parthenolide. J. Cell. Biochem..

[12] Rogakou E.P., Pilch D.R., Orr A.H., Ivanova V.S., Bonner W.M. (1998). DNA double-stranded breaks induce histone H2AX phosphorylation on serine 139. J. Biol. Chem..

[13] Li Y.H., Wang X., Pan Y., Lee D.H., Chowdhury D., Kimmelman A.C. (2012). Inhibition of non-homologous end joining repair impairs pancreatic cancer growth and enhances radiation response. PLoS ONE.

[14] Lobrich M., Shibata A., Beucher A., Fisher A., Ensminger M., Goodarzi A.A., Barton O., Jeggo P.A. (2010). gamma H2AX foci analysis for monitoring DNA double-strand break repair Strengths, limitations and optimization. Cell Cycle.

[15] Malewicz M. (2016). The role of 53BP1 protein in homology-directed DNA repair: Things get a bit complicated. Cell Death Differ..

[16] Maes K., De Smedt E., Lemaire M., De Raeve H., Menu E., Van Valckenborgh E., McClue S., Vanderkerken K., De Bruyne E. (2014). The role of DNA damage and repair in decitabine-mediated apoptosis in multiple myeloma. Oncotarget.

[17] Lassmann M., Hanscheid H., Gassen D., Biko J., Meineke V., Reiners C., Scherthan H. (2010). In Vivo Formation of gamma-H2AX and 53BP1 DNA Repair Foci in Blood Cells After Radioiodine Therapy of Differentiated Thyroid Cancer. J. Nucl. Med..

[18] Croco E., Marchionni S., Bocchini M., Angeloni C., Stamato T., Stefanelli C., Hrelia S., Sell C., Lorenzini A. (2016). DNA Damage Detection by 53BP1: Relationship to Species Longevity. J. Gerontol. A Biol. Sci. Med. Sci..

[19] Nijman S.M.B. (2011). Synthetic lethality: General principles, utility and detection using genetic screens in human cells. FEBS Lett..

[20] Brunen D., Bernards R. (2017). Drug therapy: Exploiting synthetic lethality to improve cancer therapy. Nat. Rev. Clin. Oncol..

[21] Bryant H.E., Schultz N., Thomas H.D., Parker K.M., Flower D., Lopez E., Kyle S., Meuth M., Curtin N.J., Helleday T. (2005). Specific killing of BRCA2-deficient tumours with inhibitors of poly(ADP-ribose) polymerase. Nature.

[22] Guo G.S., Zhang F.M., Gao R.J., Delsite R., Feng Z.H., Powell S.N. (2011). DNA repair and synthetic lethality. Int. J. Oral Sci..

[23] Moynahan M.E., Jasin M. (2010). Mitotic homologous recombination maintains genomic stability and suppresses tumorigenesis. Nat. Rev. Mol. Cell Biol..

[24] Shoji M., Ninomiya I., Makino I., Kinoshita J., Nakamura K., Oyama K., Nakagawara H., Fujita H., Tajima H., Takamura H. (2012). Valproic acid, a histone deacetylase inhibitor, enhances radiosensitivity in esophageal squamous cell carcinoma. Int. J. Oncol..

[25] Defoort E.N., Kim P.M., Winn L.M. (2006). Valproic acid increases conservative homologous recombination frequency and reactive oxygen species formation: A potential mechanism for valproic acid-induced neural tube defects. Mol. Pharmacol..

[26] Chen X., Wong P., Radany E., Wong J.Y. (2009). HDAC inhibitor, valproic acid, induces p53-dependent radiosensitization of colon cancer cells. Cancer Biother. Radiopharm..

[27] Adimoolam S., Sirisawad M., Chen J., Thiemann P., Ford J.M., Buggy J.J. (2007). HDAC inhibitor PCI-24781 decreases RAD51 expression and inhibits homologous recombination. Proc. Natl. Acad. Sci. USA.

[28] Koprinarova M., Botev P., Russev G. (2011). Histone deacetylase inhibitor sodium butyrate enhances cellular radiosensitivity by inhibiting both DNA nonhomologous end joining and homologous recombination. DNA Repair.

[29] Kachhap S.K., Rosmus N., Collis S.J., Kortenhorst M.S., Wissing M.D., Hedayati M., Shabbeer S., Mendonca J., Deangelis J., Marchionni L. (2010). Downregulation of homologous recombination DNA repair genes by HDAC inhibition in prostate cancer is mediated through the E2F1 transcription factor. PLoS ONE.

[30] Blattmann C., Oertel S., Ehemann V., Thiemann M., Huber P.E., Bischof M., Witt O., Deubzer H.E., Kulozik A.E., Debus J. (2010). Enhancement of radiation response in osteosarcoma and rhabdomyosarcoma cell lines by histone deacetylase inhibition. Int. J. Radiat. Oncol. Biol. Phys..

[31] Dong C., Zhang F., Luo Y., Wang H., Zhao X., Guo G., Powell S.N., Feng Z. (2015). p53 suppresses hyper-recombination by modulating BRCA1 function. DNA Repair.

[32] Feng Z.H., Scott S.P., Bussen W., Sharma G.G., Guo G.S., Pandita T.K., Powell S.N. (2011). Rad52 inactivation is synthetically lethal with BRCA2 deficiency. Proc. Natl. Acad. Sci. USA.

[33] Feng Z.H., Zhang J.R. (2012). A dual role of BRCA1 in two distinct homologous recombination mediated repair in response to replication arrest. Nucleic Acids Res..

